# Reduced Faradaic Contributions and Fast Charging of Nanoporous Carbon Electrodes in a Concentrated Sodium Nitrate Aqueous Electrolyte for Supercapacitors

**DOI:** 10.1002/ente.201900430

**Published:** 2019-06-05

**Authors:** Qamar Abbas, Bernhard Gollas, Volker Presser

**Affiliations:** ^1^ Institute for Chemistry and Technology of Materials Graz University of Technology Stremayrgasse 9 A-8010 Graz Austria; ^2^ Institute of Chemistry and Technical Electrochemistry Poznan University of Technology Bedychowo 4 60-965 Poznan Poland; ^3^ INM – Leibniz Institute for New Materials Campus D2 2 66123 Saarbrücken Germany; ^4^ Department of Materials Science and Engineering Saarland University Campus D2 2 66123 Saarbrücken Germany

**Keywords:** concentrated electrolytes, electrical double-layers, hydration, hydrogen chemisorption, supercapacitors

## Abstract

The Faradaic processes related to electrochemical water reduction at the nanoporous carbon electrode under negative polarization are reduced when the concentration of aqueous sodium nitrate (NaNO_3_) is increased or the temperature is decreased. This effect enhances the relative contribution of ion electrosorption to the total charge storage process. Hydrogen chemisorption is reduced in aqueous 8.0 m NaNO_3_ due to the low degree of hydration of the Na^+^ cation; consequently, less free water is available for redox contributions, driving the system to exhibit electrical double‐layer capacitive characteristics. Hydrogen adsorption/desorption is facilitated in 1.0 m NaNO_3_ due to the high molar ratio. The excess of water shifts the local pH in carbon nanopores to neutral values, giving rise to a high overpotential for dihydrogen evolution in the latter. The dilution effect on local pH shift in 1.0 m NaNO_3_ can be reduced by decreasing the temperature. A symmetric activated carbon cell assembled with 8.0 m NaNO_3_ exhibits a high capacitance and coulombic efficiency, a larger contribution of ion electrosorption to the overall charge storage process, and a stable capacitance performance at 1.6 V.

## 1 Introduction

Electrical double‐layer capacitors (EDLCs) are high‐power devices used in technologies such as braking energy recovery, wind‐turbine pitch control, grid power buffering, and in hybrid vehicles to enhance the lifetime of batteries.^1^ EDLCs implement nanoporous carbon electrodes with a high surface area for electrochemical charge storage via ion electrosorption.^2^ As the charges are physically accumulated at the carbon/electrolyte interface, the resulting devices exhibit a high power performance and a long cycle life.^1^, ^3^, ^4^, ^5^ The electrostatic charge storage mechanism differentiates EDLCs from pseudocapacitors, with the latter employing any Faradaic reaction that merely displays the electrical signature of a capacitor‐like charge–discharge behavior (showing a rectangular cyclic voltammogram [CV]).^6^ Both types of technologies (pseudocapacitors and EDLCs) are then categorized as supercapacitors. However, compared with batteries, supercapacitors generally exhibit a rather low energy density.^7^ The latter is dependent on the capacitance (which is controlled by the electrode material) and square dependent on the voltage (which is related to the electrochemical stability window of the electrolyte) reached by the device.

For carbon‐based supercapacitors, pH‐neutral aqueous solutions have emerged as environmental friendly and low‐cost electrolytes.^8^, ^9^, ^10^, ^11^, ^12^, ^13^, ^14^ The nonflammable character and high ionic mobility in these aqueous media give them superiority in terms of safety and power‐handling performance over traditional electrolytes (e.g., 1.0 m TEA‐BF_4_ in acetonitrile) used in supercapacitors. An important advantage of neutral aqueous electrolytes is the large potential window provided on the activated carbon electrode.^10^ The voltage window exceeding the electrochemical stability of water (*U* = 1.23 V) is due to the production of OH^−^ anions by the reduction of water at the negative carbon electrode below the thermodynamic potential, resulting in high local pH and consequently downshifting of gaseous dihydrogen evolution potential, according to the Nernst equation (*E*_H_ = −0.059 pH).^15^ Electroreduction of water in the negative potential range generates nascent hydrogen, which is chemisorbed in the porosity of carbon electrodes, according to Equation (1).

(1) $$C+xH_{2}O+xe^{-}\to 〈{\text{CH}}_{x}〉+x{\text{OH}}^{-}$$

When the amount of adsorbed hydrogen increases due to the continuous reduction of water, the hydrogen atoms combine to produce molecular (gaseous) dihydrogen.^16^, ^17^, ^18^, ^19^ However, the OH^−^ anions generated at the negative electrode are easily diluted in the presence of excessive amounts of free water in the electrolyte, thus shifting back the local pH to near neutral values according to the Le Chatelier's principle. Overall, the lower the concentration of the aqueous electrolyte, the easier is the shift of local pH from alkaline to neutral, influencing the reachable potential window of the carbon electrode. In addition, the chemisorbed hydrogen on the negative potential can be electro‐oxidized on the positive potentials, governed by Equation (2)

(2) $$〈{\text{CH}}_{x}〉+xH_{2}O\to C+xH_{3}O^{+}+xe^{-}$$

The rate of this reaction depends on the availability of free‐water molecules near the electrode surface.^20^, ^21^ So far, mostly pH‐neutral aqueous electrolytes based on alkali sulfate salts (such as Li_2_SO_4_, Na_2_SO_4_, and K_2_SO_4_) have been tested in supercapacitors.^20^, ^21^, ^22^, ^23^ One major drawback of electrolytes based on alkali sulfates is the low solubility of salts in water; for example, the maximum concentration of 2.7 m for Li_2_SO_4_, which further decreases with increasing the size of an alkali cation. In addition, neutral aqueous electrolytes based on alkali sulfates such as Li_2_SO_4_ or Na_2_SO_4_ exhibit limited conductivity values of around 70 mS cm^−1^ at a concentration of 1 m. The charging characteristics of nanoporous carbon electrodes are influenced by the ionic mobility; therefore, implementing high‐conductivity neutral aqueous electrolytes is important to obtain high‐power devices. Alkali nitrate salts are interesting candidates for aqueous electrolytes due to their high solubility, low cost, and environmental friendliness.^24^, ^25^ Sodium nitrate (NaNO_3_) offers a very high solubility in water up to 10 m at +24 °C, does not form hydrated solid phases, and the resulting electrolyte conductivity increases almost linearly with increasing concentration.^26^, ^27^

The main contribution to the deterioration of carbon/carbon cell performance in neutral aqueous electrolytes at cell voltages exceeding a value of 1.5 V under potentiostatic floating is the generation of hydrogen at the negative electrode,^22^ the oxidation of the positive carbon electrode,^23^ and the corrosion of the positive current collector.^28^, ^29^ Excessive gas generation leads to pressure build‐up inside the cell and eventually cell failure.^30^ Although the hydrogen adsorption and desorption inside the porosity of carbon remain reversible within certain potential limits, this process never is fully reversible and, therefore, consumes additional charges resulting in the reduced energy efficiency of the system at high voltages. To improve the charging performance of carbon electrode materials, the ionic species must be continuously and swiftly transported to/from the carbon/electrolyte interface within the porosity with a minimum amount of free water.^31^, ^32^ The latter factor contributes to the efficient ion packing by appropriate modification of the screening effect due to the presence of a solvent and improves in‐pore conductivity of ionic species.^33^, ^34^, ^35^, ^36^

In this work, we studied aqueous sodium nitrate (pH = 6.4) with two concentrations of 8.0 and 1.0 m NaNO_3_ as electrolytes in carbon/carbon cells. For 8.0 m NaNO_3_ (H_2_O/Na^+^ = 6.9), hydrogen gas generation at the negative carbon electrode has been remarkably reduced. In addition, the absence of free water results in a low dilution effect on local OH^−^ anion concentration, which results in a high overpotential for dihydrogen evolution. Overall, the carbon/carbon cell using 8.0 m NaNO_3_ exhibits a high capacitance, improved coulombic efficiency, energy efficiency, and predictable (continuous) evolution of resistance and capacitance during potentiostatic floating at a cell voltage value of 1.6 V.

## 2 Results and Discussion

In this work, highly microporous carbon with an average pore size of 1.1 nm was selected as the electrode material, which is suitable for the electrosorption of solvated Na^+^ (0.79 nm) and NO_3_^−^ (0.66 nm).^37^ The maximum concentration of 8.0 m NaNO_3_ was used to avoid the precipitation of salt, which can occur after preferential water adsorption in the carbon pores. In the next section, we characterize the electrochemical performance of carbon electrodes in the negative potential range in aqueous NaNO_3_ to understand the contributions of Faradaic processes. For this purpose, carbon electrodes were polarized in a three‐electrode setup using either 1.0 or 8.0 m NaNO_3_.

### 2.1 Influence of the H_2_O/Na^+^ Ratio on the Electrochemical Hydrogen Chemisorption

**Figure 1:**
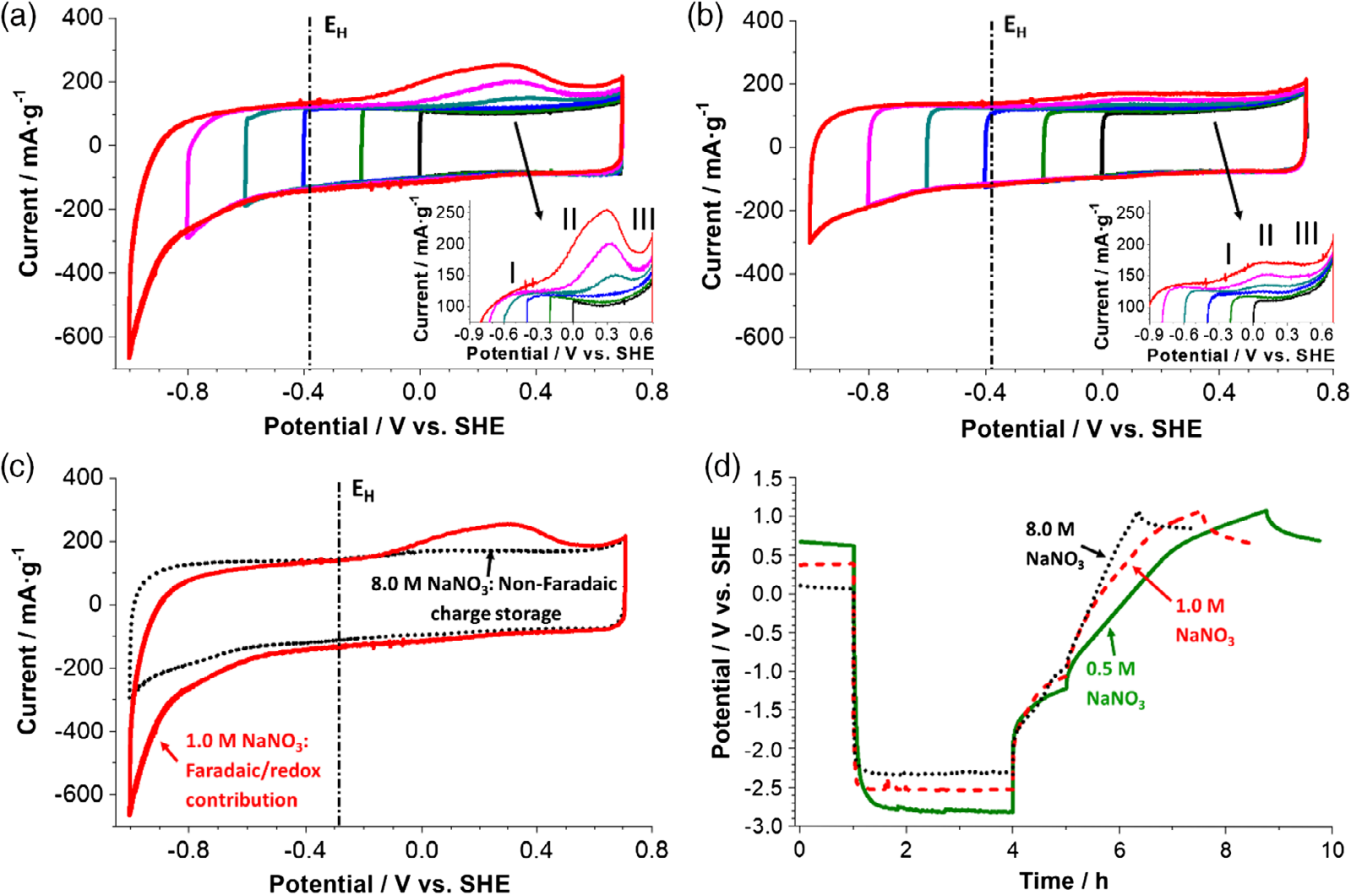
Three‐electrode cell investigation using cyclic voltammetry (2 mV s^−1^) on the activated carbon (DLC Supra30) working electrode by a stepwise decrease in potential down to −1.0 V versus SHE: a) in 1.0 m NaNO_3_, b) in 8.0 m NaNO_3_, and c) comparison of 1.0 m NaNO_3_ (full line) and 8.0 m NaNO_3_ (dotted line) down to −1.0 V versus SHE. d) Galvanostatic charging profile of the carbon electrode using aqueous 0.5 m NaNO_3_ (full green line), 1.0 m NaNO_3_ (dashed red line), and 8.0 m NaNO_3_ (dotted black line) at 2 A g^−1^, followed by a 1 h resting period, and then discharging at 0.5 A g^−1^. *E*_H_: theoretical gaseous dihydrogen evolution potential.

Cyclic voltammetry (Figure 1) shows the hydrogen adsorption and desorption at the activated carbon working electrode in aqueous 1.0 and 8.0 m NaNO_3_ in a three‐electrode cell. The stepwise decrease of potentials in Figure 1a reveals the well‐known gradual increase of negative currents due to the electroreduction of water to −1.0 V versus standard hydrogen electrode (SHE). At each step, the current increases after exceeding the thermodynamic potential of water reduction (*E*_H_), indicating that the water‐reduction intensity increases as the potential reaches more negative values.

At −1.0 V versus SHE at reduction current values around −650 mA g^−1^, the electroreduction of water causes maximum hydrogen generation at the carbon electrode. This nascent hydrogen is mainly weakly chemisorbed inside carbon nanopores^19^, ^38^ until reaching a saturation level, where atomic hydrogen combines to form molecular hydrogen. Upon further decrease in the negative potential, the molecular dihydrogen bubbles evolve and leave the carbon pore network, as can be inferred from the oscillation of our measured CVs.^17^ Moreover, the production of OH^−^ anions due to the electroreduction of water increases the pH inside the porosity of the carbon electrode up to pH = 14, estimated from the oscillation onset potential −0.8 V versus SHE. This effect provides an important overpotential to reach high voltage values in carbon/carbon setup of supercapacitors.

The reverse scan in the positive potential direction after polarization to negative potentials demonstrates a nearly constant current region at about −0.5 to −0.35 V versus SHE, followed by the electro‐oxidation of adsorbed hydrogen that starts after the thermodynamic reduction potential starting from −0.35 V versus SHE and reaching a maximum potential at about 0.3 V versus SHE. The weaker chemical interaction of generated hydrogen with the surface of activated carbon facilitates the electro‐oxidation of hydrogen for positive polarization in neutral aqueous electrolytes at 1.0 m concentration.^21^ The intensity of the hydrogen desorption peak increases with the decreasing potential limit, which demonstrates that the hydrogen adsorption and desorption are highly reversible with a 90% charging/discharging energy efficiency for our carbon electrode operating in 1.0 m NaNO_3_. The excess availability of free‐water molecules favors the adsorption and desorption reactions per Equation (1) and (2). After a maximum of hydrogen desorption, a drop in current suggests that the hydrogen stored in the deep porosity is also desorbed. A further positive polarization yields an increase in current, indicating the onset of oxidation of the carbon electrode material. The inset in Figure 1a shows the hydrogen oxidation curve enlarged at three different sites of hydrogen storage on the carbon electrode marked by peaks I, II, and III.

The electrochemical response of 8.0 m NaNO_3_ differs from that of 1.0 m NaNO_3_. As shown in Figure 1b, the polarization of activated carbon to −1.0 V versus SHE yields in 8.0 m NaNO_3_ a much lower cathodic current (−300 mA g^−1^). Seemingly, less hydrogen is stored within the carbon nanopores at a higher molar concentration, where the molar ratio of H_2_O/Na^+^ is only 6.9 compared with 55.6 for 1.0 m NaNO_3_. This significantly lowers the available number of water molecules that can partake in the reduction reaction in the case of 8.0 m NaNO_3_, and therefore, the amount of generated hydrogen is reduced. Accordingly, we observed the absence of any oscillation of the CV when scanning to −1.0 V versus SHE; this is in accordance with the absence of significant hydrogen gas evolution. The reverse scan also shows that the electro‐oxidation of stored hydrogen is also reduced in 8.0 m NaNO_3_; the energy efficiency related to hydrogen chemisorption and desorption regions of charge/discharge curve in 8.0 m NaNO_3_ is very low (≈30% down to  1.0 V vs SHE). Scanning from negative to positive polarization, we see in the inset of Figure 1b that at a high molar strength, the current peak in the region (II) is strongly suppressed (compared with a low molar strength), which is probably due to the different natures of chemical bonding of hydrogen in this system oxidized from carbon sites.

Figure 1c shows that the charges are mainly stored in the electrical double‐layer of a carbon electrode in 8.0 m NaNO_3_, whereas due to the low number of ionic species, the electrochemical reduction of water is favored in case of 1.0 m NaNO_3_ giving rise to enhanced Faradaic processes down to −1.0 V versus SHE. As can be inferred from the electrochemical signature, the main contribution to the charging current in aqueous 8.0 m NaNO_3_ relates to the electric double‐layer capacitance. In contrast, a significant contribution to the charging current in 1.0 m NaNO_3_ is due to the Faradaic processes related to reversible electrochemical hydrogen storage inside the carbon nanopores at negative potential regions down to −1.0 V versus SHE. During desorption of hydrogen, the absence of free water hinders the electro‐oxidation in 8.0 m NaNO_3_, which in case of 1.0 m NaNO_3_ is favored due to the presence of excess free water (Equation (2)).

The data in Figure 1d for 0.5, 1.0, and 8.0 m NaNO_3_ confirm that when the carbon electrode is charged galvanostatically at high negative potentials, the electrochemical hydrogen storage capacity decreases with increasing electrolyte concentration. Noticeably, the negative potential reach of the electrode increases with decreasing the electrolyte concentration, suggesting that more energy is needed to reduce water and chemisorb hydrogen with the diluted electrolyte. This effect suggests that water bonded to Na^+^ cations is probably reduced at the pore entrance. Therefore, in diluted electrolytes where ionic species are less in number, the ionic migration is highly dependent on electrochemical polarization. In contrast, when the electrolyte concentration increases, the solvated ionic species easily reach the porosity of the carbon electrode, requiring less energy, and therefore, a higher reduction potential is enough. For the 0.5 m NaNO_3_ (Figure 1d), after charging for 3 h, the straight‐line during discharge aligns with a capacitance mainly caused by electrical double‐layer charging until reaching an inflection point (≈0.5 V vs SHE), which suggest the desorption of hydrogen. Further polarization results in the emergence of a plateau that marks the onset of oxidation of the carbon electrode material. When increasing the ion concentration to 1.0 m NaNO_3_, we see a discharge curve with more contribution from the electrical double‐layer charging, which reaches a maximum for the highest electrolyte concentration. Overall, with increasing concentration of aqueous NaNO_3_, the Faradaic contributions related to hydrogen chemisorption are remarkably reduced on a carbon electrode charged at negative potentials.

**Figure 2:**
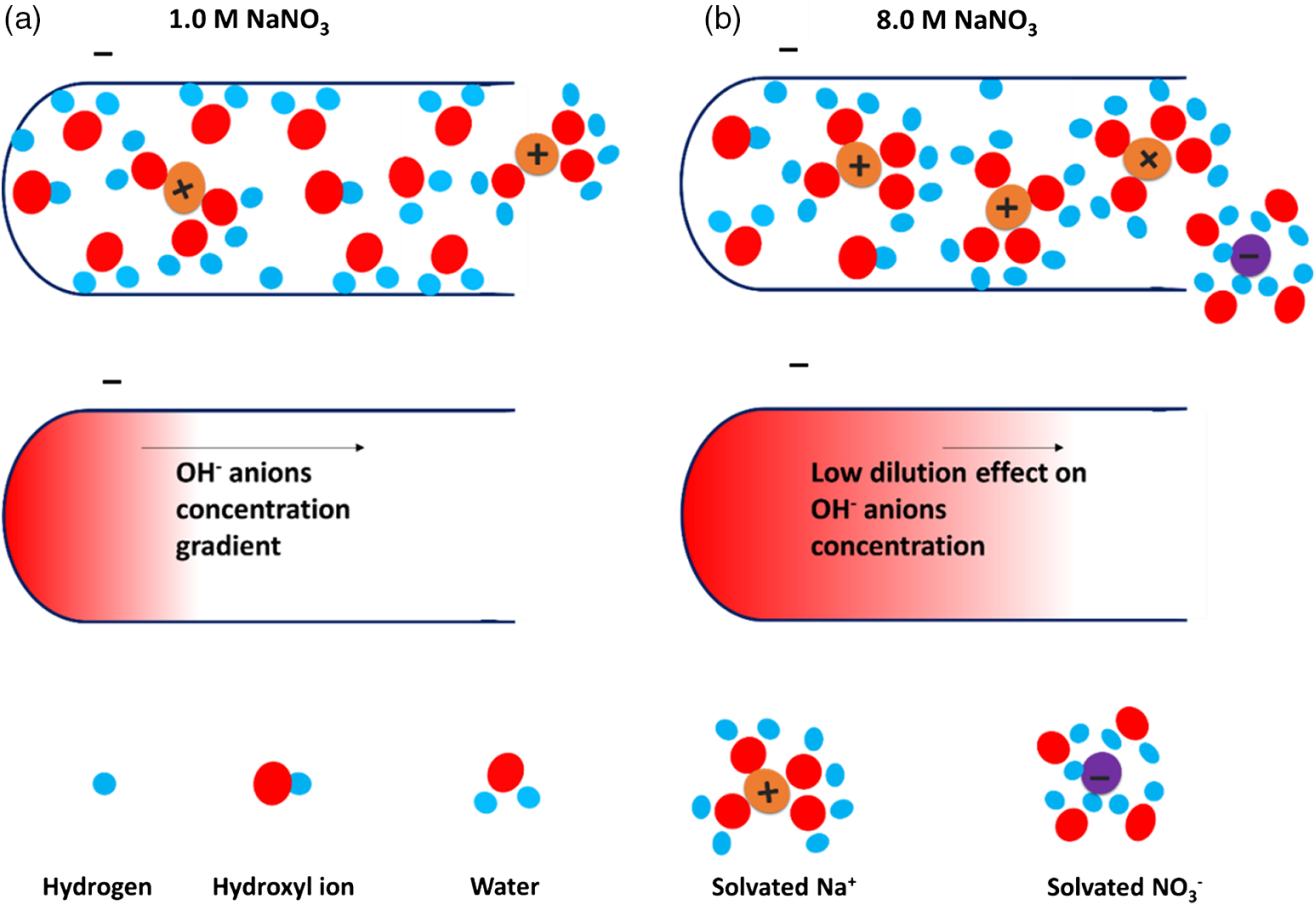
Schematic illustration of hydrogen chemisorption and OH^−^ anion generation inside a pore under negative polarization. Due to the excess of free‐water available in a) 1.0 m NaNO_3_, the OH^−^ anions are easily diluted due to the diffusion of water from the bulk electrolyte, whereas the concentration of OH^−^ anions remains almost unchanged inside the pore (at an alkaline pH) b) when concentrated 8.0 m NaNO_3_ is used as an electrolyte.

Figure 2 shows the two ideal cases where the local pH inside the porosity of carbon is easily restored back to neutral values under the dilution effect of OH^−^ anions. The local pH inside the porosity would be still alkaline (pH ≈ 14) in case of 8.0 m NaNO_3_, thus providing a high overpotential for the negative potential region. The saturation of porosity with ionic species is an important parameter that controls the capacitive behavior of the nanoporous carbon electrode. In 1.0 m NaNO_3_, ionic species are easily accommodated for ion electrosorption. For the diluted electrolyte, water molecules form an oriented layer at the pore wall. In the case of a concentrated solution, the solvated cations are in contact with the electrode, and fewer free‐water molecules are attached to the pore wall, thus reducing the Faradaic contributions. These findings demonstrate that high concentration of 8.0 m NaNO_3_ is favorable for a supercapacitor application when the goal is to achieve a high ion electrosorption contribution to the total charging current.

### 2.2 Improved Electrochemical Performance of Two‐Electrode Cells in 8.0 m NaNO_3_

**Figure 3:**
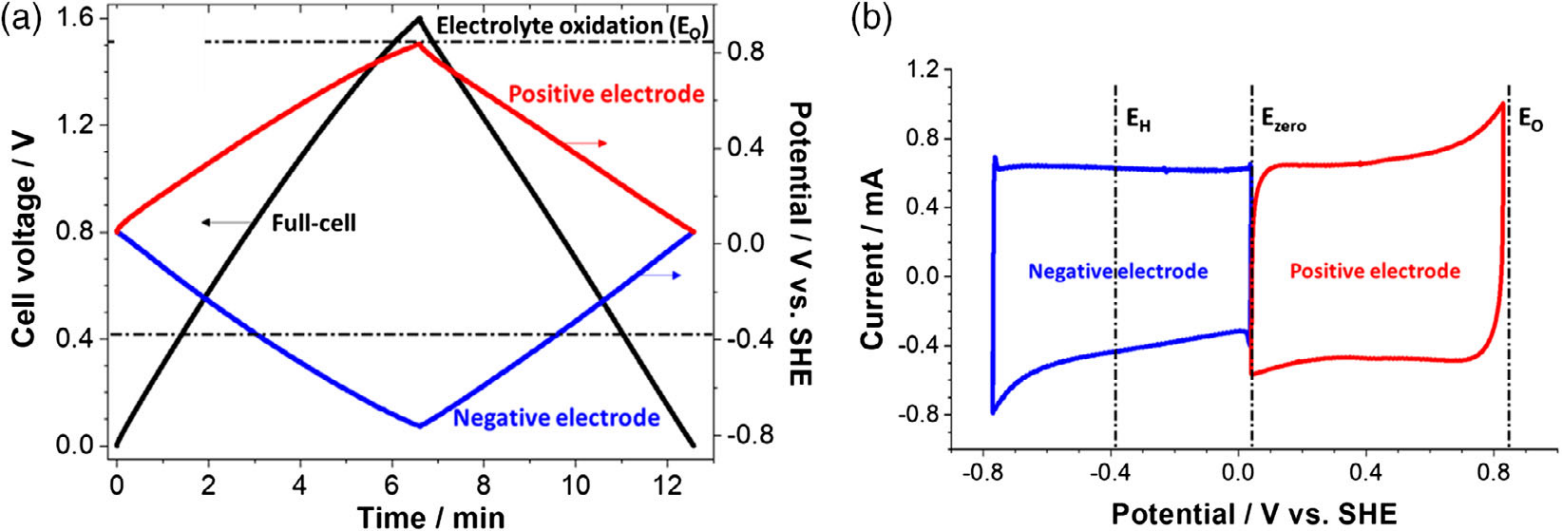
Potential limits of positive and negative electrodes in a two‐electrode cell with a reference setup using 8.0 m NaNO_3_ from 0 to 1.6 V: a) galvanostatic charge/discharge curves at 0.2 A g^−1^, b) CVs at 1 mV s^−1^ according to the estimated limits in the positive and negative potential range (dashed horizontal and vertical lines indicate the thermodynamic potential limits of water oxidation [*E*_O_] and reduction [*E*_H_]).

**Figure 4:**
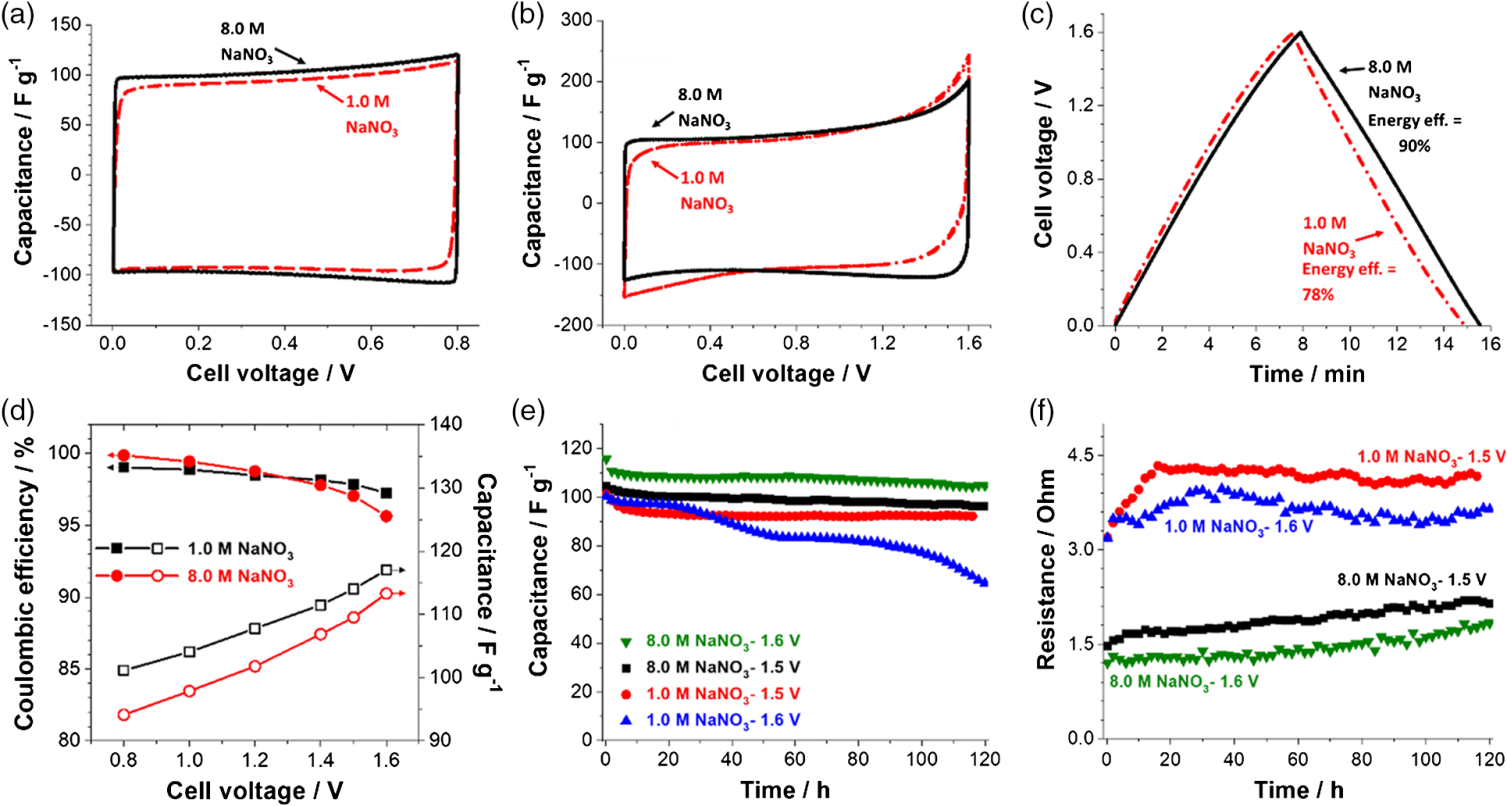
Comparison of electrochemical performance of carbon/carbon capacitors in 1.0 m NaNO_3_ (dashed red line) and 8.0 m NaNO_3_ (full black line): a) CVs (2 mV s^−1^) up to 0.8 V, b) CVs (2 mV s^−1^) up to 1.6 V, c) GCPL (0.2 A g^−1^) up to 1.6 V, d) capacitance (expressed per mass of one electrode) and coulombic efficiency for different voltage values up to 1.6 V at 0.2 A g^−1^, e) evolution of capacitance, and f) evolution of cell resistance during potentiostatic floating at 1.5 and 1.6 V.

Carbon/carbon full‐cells equipped with a reference electrode are polarized in 8.0 m NaNO_3_ up to 1.6 V galvanostatically at a specific current of 0.2 A g^−1^, and the potential ranges of positive and negative electrodes are established as shown in Figure 3a. Both positive and negative electrodes exhibit a symmetric charge/discharge behavior. Hence, in a symmetric carbon/carbon cell where the voltage is equally assigned for the positive and negative electrodes, the capacitance of the cell is estimated by Equation (3)

(3) $$\frac{1}{C_{\text{cell}}}=\frac{1}{C_{+}}+\frac{1}{C_{-}}$$

where *C* is the capacitance, which can be expressed per active mass of one electrode or two electrodes in such a cell.^39^ The negative carbon electrode exhibits a squarish CV (Figure 3b) that is typical for a capacitor‐like behavior associated with ion electrosorption. However, at a low negative potential of about −0.8 V versus SHE, an increase of the cathodic current is caused by the contributions from electroreduction of water, but there is no response on the reverse scan for this contribution. Although the negative electrode in 8.0 m NaNO_3_ operates below the thermodynamic reduction potential limit, there are only very small seemingly Faradaic contributions related to the generation of hydrogen at this electrode. Simultaneously, the positive carbon electrode in aqueous NaNO_3_ operates below the thermodynamic water oxidation potential limit exhibiting a square‐shaped CV with small Faradaic contributions at the highest potential value at 0.81 V versus SHE. Nevertheless, these Faradaic contributions on both positive and negative electrodes in 8.0 m NaNO_3_ due to slight oxidation and reduction of aqueous NaNO_3_ do not influence the evolution of electrochemical characteristics such as capacitance and resistance of the cell to a great extent as shown in Figure 4. The evolution of an electrochemical response of the carbon electrode on positive potentials was also investigated; for that, the electrode was polarized to 1.05 V versus SHE, and a sudden increase of the charging current was observed, which indicated the onset of carbon oxidation (Figure S1, Supporting Information).

The galvanostatic charge/discharge curves at 1.6 V of full‐cells operating either at 1.0 or 8.0 m NaNO_3_ are provided in Figure 4c, and Table 1 lists the corresponding energy‐efficiency values. The lower energy efficiency of 83% and lower symmetry of the charge/discharge curves obtained in aqueous 1.0 m NaNO_3_ reflect the occurrence of Faradaic processes as the cell voltage reaches 1.6 V. In contrast, the galvanostatic charge/discharge curve for operation in aqueous 8.0 m NaNO_3_ presents a more symmetric triangular shape, which translates to a high energy efficiency of 90% at 1.6 V. From Table 1, we also see that the higher efficiency is equally expressed at lower cell voltages.

**Table 1:** Energy efficiency (%) of carbon/carbon capacitors with 8.0 and 1.0 m NaNO_3_ at various cell voltage values

	0.8 V	1.0 V	1.2 V	1.4 V	1.5 V	1.6 V
8.0 m NaNO_3_	94.1	93.5	92.8	92.1	91.3	90.0
1.0 m NaNO_3_	90.1	89.3	87.9	87.0	85.3	83.0

The capacitance (estimated from the discharge time and expressed per active mass of one electrode) and coulombic efficiency values versus voltage of capacitors using both concentrations of NaNO_3_ are shown in Figure 4d. Up to 1.6 V, the symmetric full‐cells operated in aqueous 8.0 m NaNO_3_ demonstrate a higher capacitance of 117 F g^−1^ and higher coulombic efficiency of 97% compared with the cell using 1 m NaNO_3_ exhibiting a capacitance of 107 F g^−1^ and coulombic efficiency of 94%.

We further quantified the performance stability of symmetric full‐cells using either aqueous 1.0 or 8.0 m NaNO_3_ via potentiostatic floating at cell voltages of 1.5 and 1.6 V.^40^, ^41^ The capacitance values (Figure 4e) in cells using 8.0 m NaNO_3_ show a constant trend and simultaneous low constant resistance evolution and, thereby, exhibit a very stable performance over time even at 1.6 V. When using 1.0 m NaNO_3_ instead, we see a stable capacitance performance only at 1.5 V and a significant and continued decrease of the charge storage ability over time at 1.6 V. The corresponding resistance values during potentiostatic floating are shown in Figure 4f. The data show the overall higher resistance at low molar concentration, but both concentrations show rather stable resistance development.

**Figure 5:**
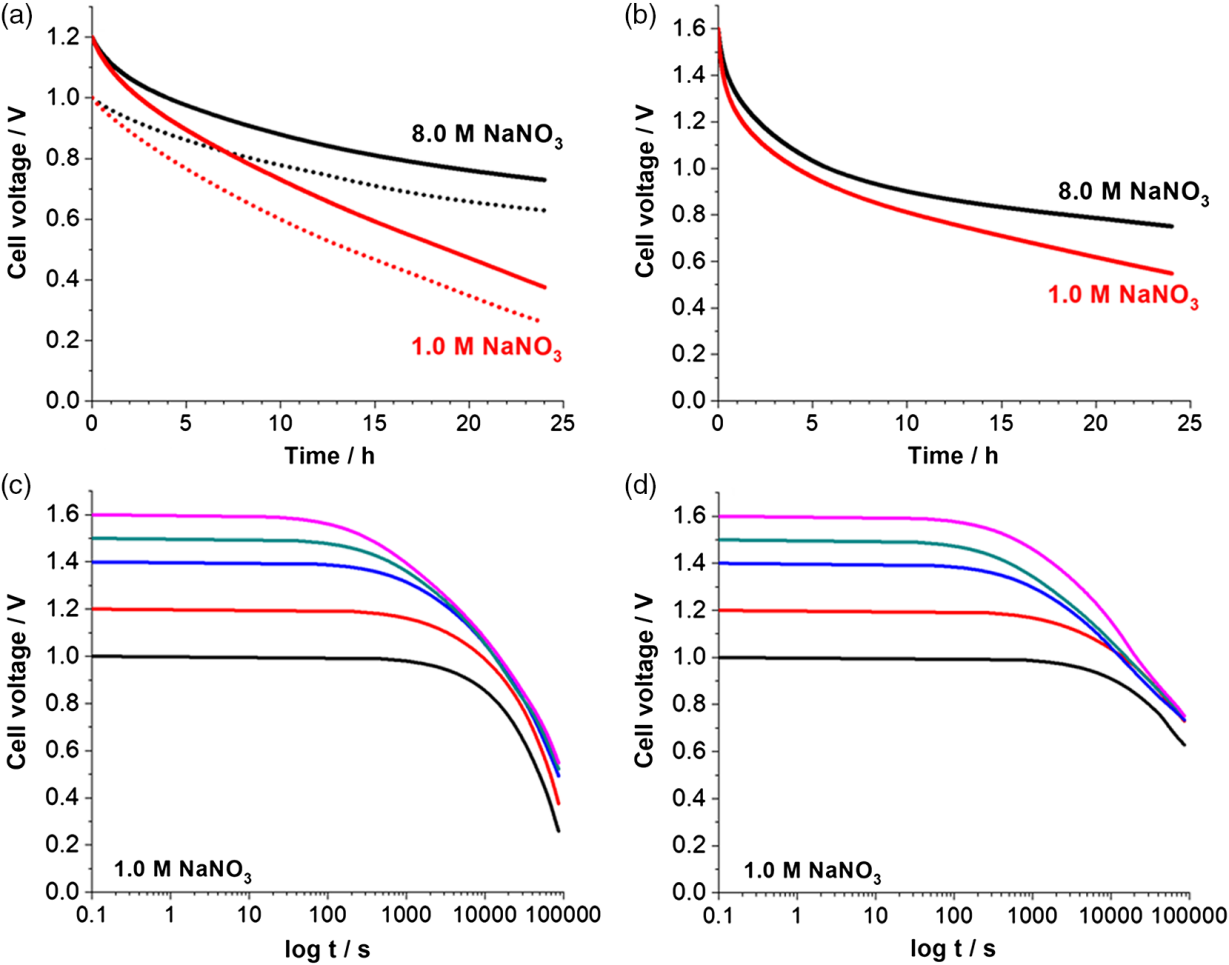
a) Voltage‐decay profiles for full‐cells in 8.0 and 1.0 m NaNO_3_ up to 1.0 V (dotted lines) and 1.2 V (full lines) and b) up to 1.6 V cell voltage. c) Log(time) versus cell voltage with 1.0 m NaNO_3_ and d) log(time) versus cell voltage for a cell operated with 8.0 m NaNO_3_.

Figure 5 shows the self‐discharge after 8 h of holding at the maximum voltage and evolution of open‐circuit voltage for 24 h. After charging to a cell voltage of 1.0 or 1.2 V (Figure 5a), the capacitor with 1.0 m NaNO_3_ shows a larger self‐discharge (Δ*U*_1.0_ = 0.74 V, Δ*U*_1.2_ = 0.83 V) compared with 8.0 m NaNO_3_ (Δ*U*_1.0_ = 0.38 V, Δ*U*_1.2_ = 0.47 V). When charged to 1.6 V (Figure 5b), cells with both concentrations exhibit high self‐discharge, but the cell with 8.0 m NaNO_3_ shows lower Δ*U*_1.6_ = 0.84 V than the one with 1.0 m NaNO_3_ (Δ*U*_1.6_ = 1.07 V). The plots of voltage versus log *t* in Figure 5c,d when using 1.0 or 8.0 m NaNO_3_ show a more activation‐controlled mechanism at all voltages in the former case.^42^, ^43^, ^44^, ^45^ The discharge processes are more diffusion‐controlled at low voltages for the cell in 8.0 m NaNO_3_, which shift to slightly activation‐controlled ones at higher voltages.

### 2.3 Effect of Decreasing Temperature on Hydrogen Storage and Charging Characteristics

**Figure 6:**
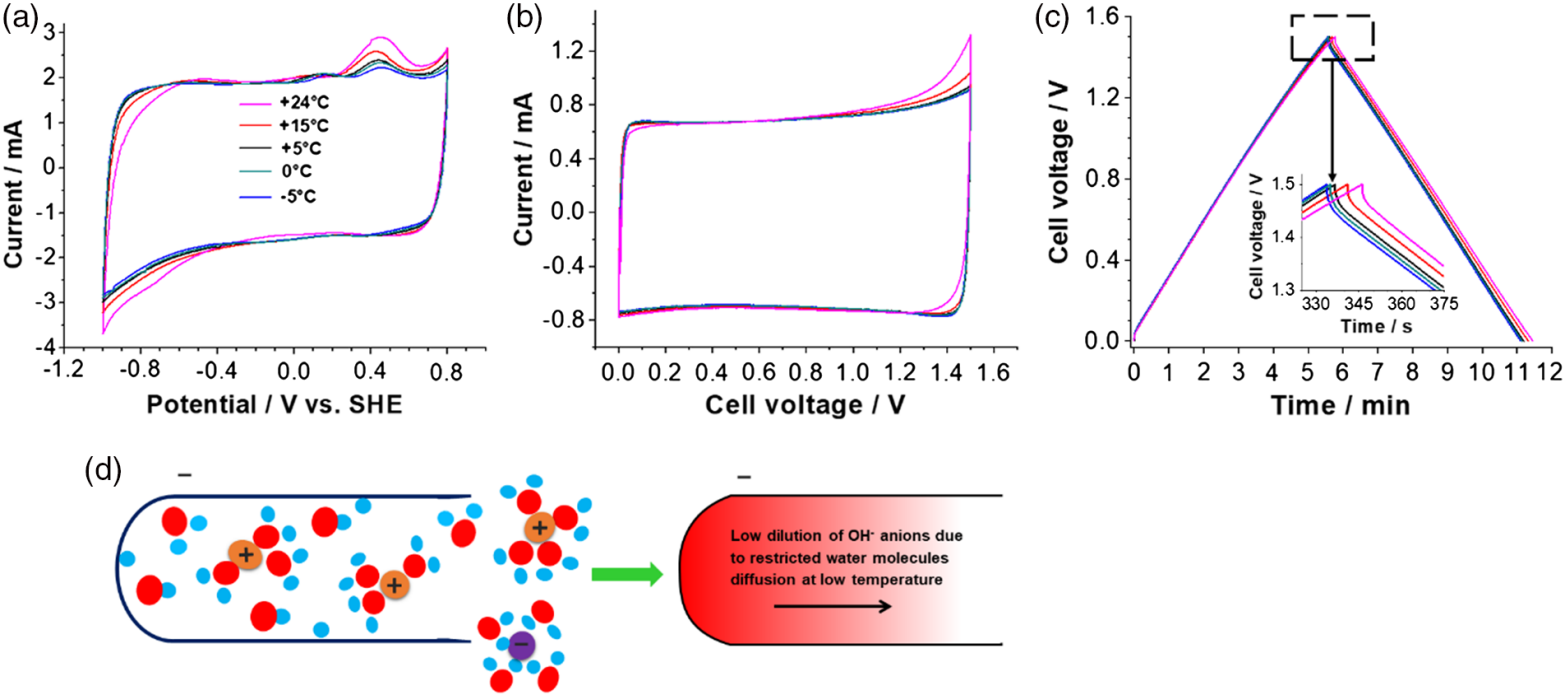
a) Three‐electrode (half‐cell) data using cyclic voltammetry (2 mV s^−1^) of the activated carbon working electrode at −1.0 V versus SHE in 1.0 m NaNO_3_, b) cyclic voltammetry at 2 mV s^−1^, and c) galvanostatic charge/discharge cycling at 0.2 A g^−1^ by a stepwise decrease in temperature from +24 to −5 °C. d) Schematic illustration of electrochemical hydrogen storage and the formation of OH^−^ anions inside a carbon nanopore under negative polarization at a low temperature.

To better understand the proposed dependency of electrochemical hydrogen storage on the availability of free water, three‐electrode cell investigations were conducted on an activated carbon electrode in 1.0 m NaNO_3_ at different temperatures. As the voltammetry response of a full cell in 8.0 m NaNO_3_ was unchanged from +24 to −5 °C (Figure S2, Supporting Information), half‐cell measurements were carried out only in 1.0 m NaNO_3_. Figure 6a shows the CVs of a half‐cell when sweeping from +0.8 to −1.0 V versus SHE using 1.0 m NaNO_3_. We monitored the hydrogen adsorption and desorption with a stepwise decreasing temperature from +24 to −5 °C. With the decrease in temperature, the adsorption pattern slightly changes due to the reduction of water below the thermodynamic potential limit at pH = 6.4 (−0.38 V versus SHE). In addition, the desorption peak at about 0.4 V versus SHE is also reduced with decreasing temperature, which suggests that oxidation of hydrogen via Equation (2) is energetically less favored at low temperatures.^17^

We further tested symmetric full‐cells at different temperatures (Figure 6b,c), and the electrochemical signatures show that the reduced hydrogen reactivity of half‐cells also reduced the Faradaic current with reduced temperatures during forward scanning near the maximum cell voltage of 1.6 V.

Figure 6d shows the processes occurring at the fluid–solid interface of the carbon nanopore under negative polarization in 1.0 m NaNO_3_ at low temperatures. Due to the restricted movement of water molecules, the OH^−^ anions are accumulated inside the pores leading to a high local pH and consequently enhanced overpotential. Overall, upon progressively decreasing the operational temperature from +24 to −5 °C, the hydrogen chemisorption decreases and the desorption peak is reduced.

## 3 Conclusions

Our work has shown a strong dependency of ion concentration and temperature on electrochemical hydrogen storage in an aqueous NaNO_3_ electrolyte. The Faradaic contributions on an activated carbon electrode in a negative potential range using 8.0 m NaNO_3_ are greatly reduced, where the hydrogen adsorption and desorption are dramatically suppressed. We can explain this behavior per the high ionic strength and less availability of reduceable free‐water molecules due to strong H_2_O—Na^+^ bonding in concentrated aqueous NaNO_3_. This contrasts with the situation when a nanoporous carbon electrode is polarized in 1.0 m NaNO_3_, where a large amount of hydrogen is produced and stored inside the porosity of carbon. Moreover, we have seen a high performance stability of capacitance and cell resistance values during voltage floating at 1.6 V in aqueous 8.0 m NaNO_3_ electrolyte.

## 4 Experimental Section

4.1

4.1.1

##### Materials and Material Characterization

NaNO_3_ was purchased from Sigma Aldrich (>99%) and dried at +110 °C for 12 h under vacuum before dissolving in deionized water to prepare solutions of two concentrations (1.0 and 8.0 m). The conductivity of the bulk aqueous solutions was determined with an ELMETRON CPC‐501 conductometer (with cell calibration in standard 0.1 m KCl solution). The conductivity of 1.0 m NaNO_3_ was found to be 72 and 168 mS cm^−1^ for 8.0 m NaNO_3_ (Figure S3, Supporting Information), and both electrolytes had a neutral pH of 6.4.

The activated carbon used in this work was DLC Supra 30 from Norit, and additional material characterization data are provided in the Supporting Information. With a sorption isotherm typical for micropore‐dominated materials (Figure S4a, Supporting Information), the material had a specific surface area of 1344 m^2^ g^−1^, a micropore volume of 0.68 cm^3^ g^−1^, and a mesopore volume of 0.11 cm^3^ g^−1^ (Figure S4b, Supporting Information). Structurally, the activated carbon consisted of incompletely graphitic carbon, as can be seen from the D‐ and G‐modes observed at 1344 and 1602 cm^−1^, respectively, observed via Raman spectroscopy (Figure S5, Supporting Information). Thermogravimetric analysis coupled with mass‐spectrometry (Figure S6, Supporting Information) showed low amounts of H_2_O, CO, and CO_2_, which indicated the presence of few surface functional groups.

##### Electrode Preparation and Electrochemical Characterization

For the electrochemical investigations, electrodes were prepared by mixing 90 mass% of activated carbon, 5 mass% of carbon black Super C65 (Imerys) as a conductive additive, and 5 mass% of polytetrafluoroethylene (60 mass% suspension in water; Sigma‐Aldrich) as a binder in isopropanol. The dough obtained after mixing these components was rolled to a 200 µm thick sheet, which was dried at +120 °C for 12 h under vacuum, and disks were punched for direct use as electrodes. We used Swagelok‐type three‐electrode cells for half‐cell measurements with nanoporous carbon as a working electrode, carbon electrode (the same material as a working electrode but with the mass twice higher) as a counter electrode, stainless steel current collectors, and a silver/silver chloride (Ag/AgCl in KCl_sat_, *E* = +0.197 V vs SHE) as a reference electrode. For hydrogen storage investigations at various temperatures, an identical three‐electrode cell was constructed using 1.0 m NaNO_3_ and placed inside a Binder climatic chamber MK‐53 (E2), and the temperature was scanned from +24 to −5 °C. The potential in three‐electrode cells was decreased stepwise in the negative direction at a scan rate of 2 mV s^−1^ until reaching −1.0 V versus SHE.

Two‐electrode cells with and without a reference electrode were constructed also in a Swagelok‐type device using 1.2 cm diameter current collectors, 1.0 cm diameter disks as working and counter electrodes sandwiching a glass microfiber separator (Whatman GF/A, 260 µm thick), and 8.0 m aqueous NaNO_3_ electrolyte (degassed for 15 min under reduced pressure at +24 °C). The current collectors were made from stainless steel 316L. The mass of the positive carbon electrode (*m*_+_) was kept at 1.5 times the mass of the negative electrode (10–12 mg; *m*_−_) to balance the charges (*q*_+_ and *q*_−_) by considering the specific capacitance (*C*_+_ and *C*_−_) according to Equation (4) and (5).

(4) $$q_{+}=q_{-}$$

(5) $$m_{+}\times C_{+}\times \Delta E_{+}=m_{-}\times C_{-}\times \Delta E_{-}$$

The potential ranges of activated carbon electrodes (Δ*E*) in a two‐electrode cell using 8.0 m NaNO_3_ with a reference were determined up to a cell voltage *U* = 1.6 V by galvanostatic cycling with potential limitation (GCPL, 0.2 A g^−1^), and also the corresponding CVs (1 mV s^−1^) were measured. The carbon/carbon symmetric cells were investigated by CV (2 mV s^−1^) and GCPL (0.2 A g^−1^, the current expressed per active mass of one electrode) up to a cell voltage of *U* = 1.6 V. The current and capacitance values were normalized to the mass of activated carbon, which was 90% of the total electrode mass. All electrochemical measurements were realized with a VMP3 potentiostat/galvanostat (Bio‐Logic Instruments). The gravimetric capacitance *C* was calculated from galvanostatic discharge data and expressed per active mass of one electrode (F g^−1^) using Equation (6)

(6) $$C=\frac{2I}{(dU/dt)\times m}$$

where *I* is the current (A), d*U/*d*t* is the slope of the discharge curve (V s^−1^), *m* is the average mass of activated carbon in one electrode (g). The energy efficiency was estimated by the ratio of the integrated surface area under the galvanostatic discharge/charge curves, and the coulombic efficiency from the ratio of discharge time over the charge time (*t*_d_/*t*_c_).

We used potentiostatic floating as an accelerated method to estimate aging at +24 °C. The cells were charged to 1.6 V and maintained at this voltage for 2 h; thereafter, five galvanostatic charge/discharge cycles were carried out at a specific current of 1.0 A g^−1^. The capacitance was quantified from the fifth discharge and the equivalent series resistance (ESR) from the voltage drop between the fifth charge and discharge when reversing the sign of current. A total of 60 floating/galvanostatic periods were applied, representing a cumulated floating time of 120 h. The voltage profile of the full cell during open circuit was monitored for 24 h after a voltage hold for 8 h at 1.0 or 1.6 V.

## Conflict of Interest

The authors declare no conflict of interest.

## Supporting information

Supplementary
