# Supplementary material for: Reduced Faradaic Contributions and Fast Charging of Nanoporous Carbon Electrodes in a Concentrated Sodium Nitrate Aqueous Electrolyte for Supercapacitors
Source: Energy Technol (Weinh). 2019 Jun 5;7(9):1900430. doi: 10.1002/ente.201900430 (PMC6774286; doi:10.1002/ente.201900430)
Supplement: Supplementary file 1 — Supplementary [file ENTE-7-na-s001.docx]

Supporting Information

Reduced Faradaic contributions and fast charging of nanoporous carbon electrodes in a concentrated sodium nitrate aqueous electrolyte for supercapacitors

*Qamar Abbas ^[a,b,*]^, Bernhard Gollas ^[a]^, Volker Presser ^[c,d]^*

Dr. Qamar Abbas, Prof. Bernhard Gollas

[a] Institute for Chemistry and Technology of Materials, Graz University of Technology, Stremayrgasse 9, A-8010 Graz, Austria
E-mail: [qamar.abbas@tugraz.at](mailto:qamar.abbas@tugraz.at)

Dr. Qamar Abbas

[b] Institute of Chemistry and Technical Electrochemistry, Poznan University of Technology, Bedychowo 4, 60-965 Poznan, Poland

Prof. Volker Presser

[c] INM – Leibniz Institute for New Materials, Campus D2 2, 66123 Saarbrücken, Germany

[d] Department of Materials Science and Engineering, Saarland University, Campus D2 2, 66123 Saarbrücken, Germany

1. **Supporting electrochemical characterization**


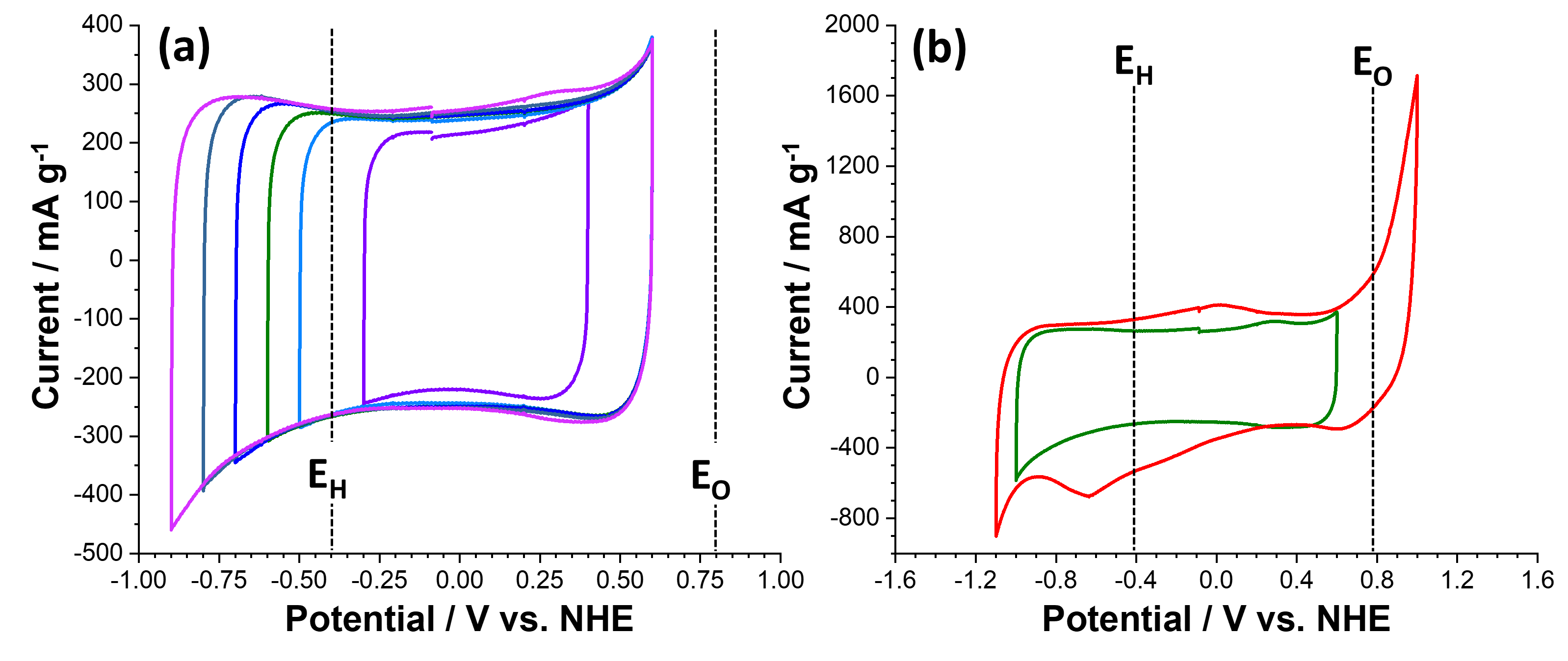


**Figure S1**. (a) Cyclic voltammograms at 2 mV∙s^-1^ on the activated carbon (DLC Supra30) working electrode in 8.0 M NaNO_3_ by stepwise decreasing of potential to -0.8 V vs. SHE and (b) by increasing the values in the positive potential range.


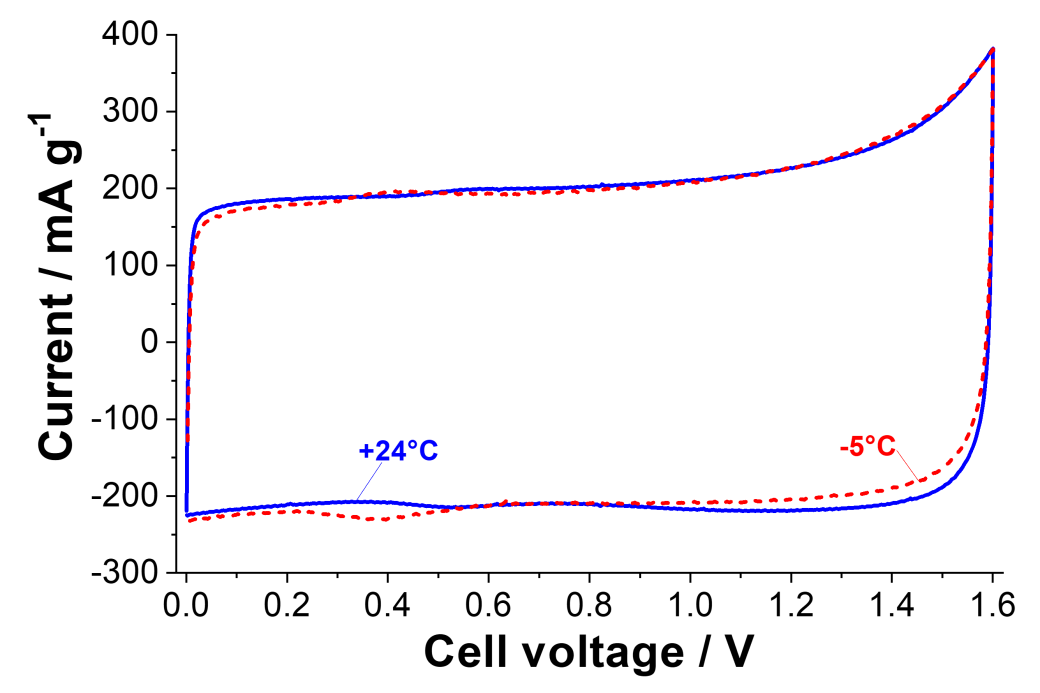


**Figure S2**. Cyclic voltammograms (2 mV∙s^-1^) of a symmetric full-cell in 8.0 M NaNO_3_ up to 1.6 V at +24°C and -5°C.

1. **Concentration-dependent conductivity of aqueous NaNO_3_**

**
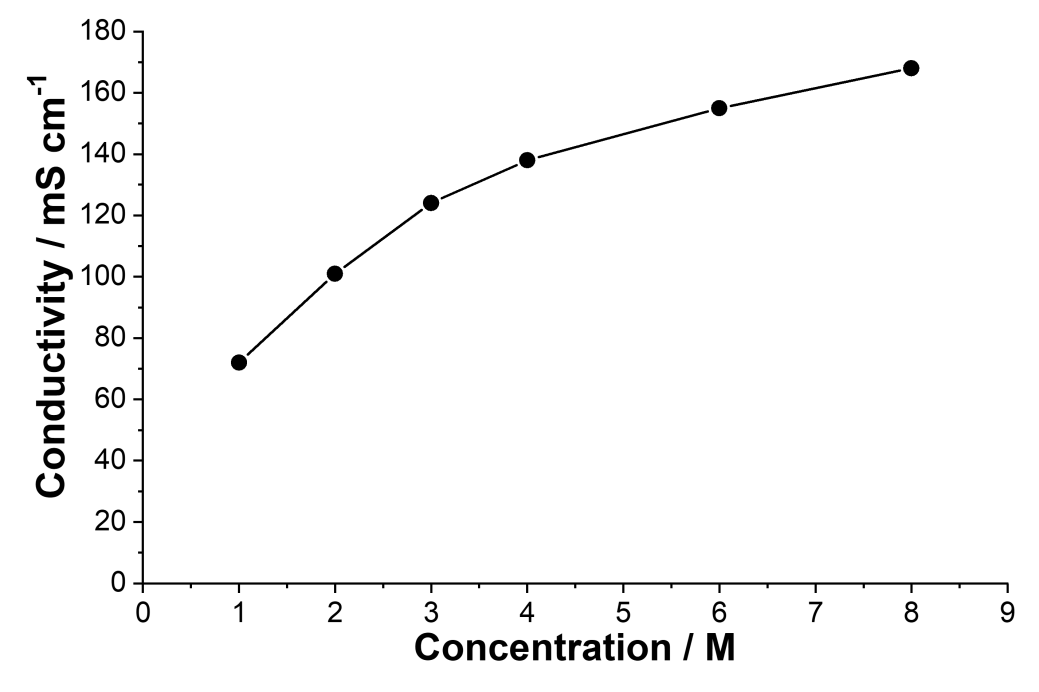
**

**Figure S3**. Conductivity profile of aqueous NaNO_3_ electrolyte between 1.0 M and 8.0 M measured at +24°C.

1. **Material characterization of Norit DLC Supra 30**


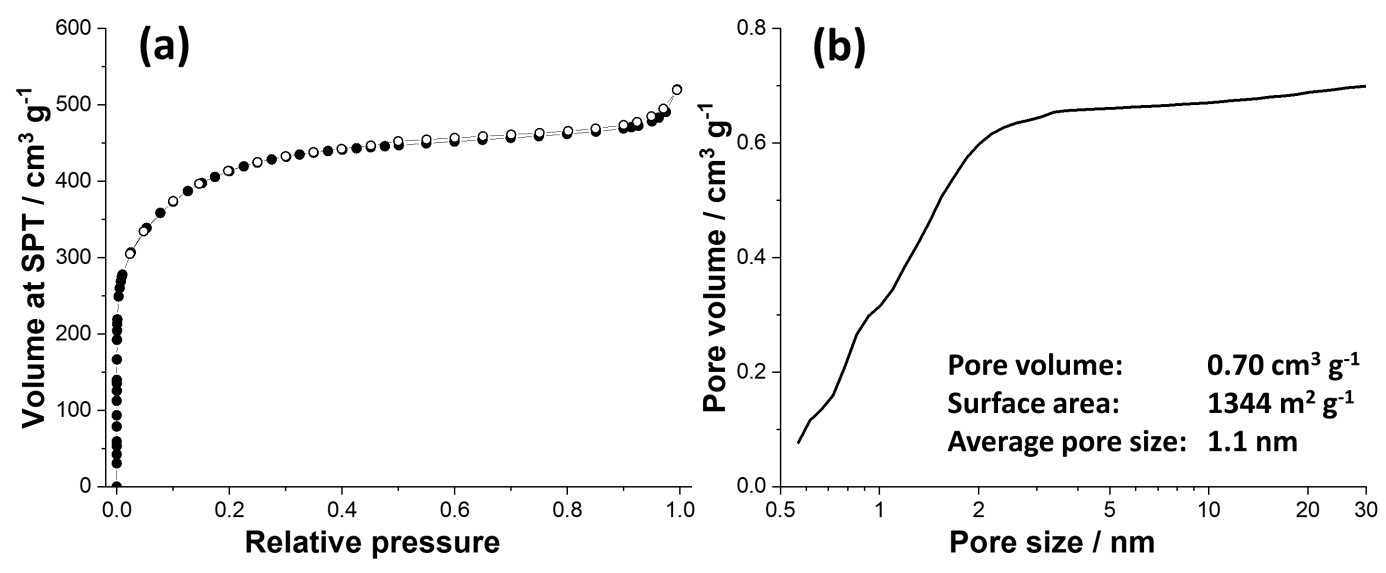


**Figure S4**. Nitrogen gas sorption data obtained at -196°C with a Quantachrome iQ system. (a) Nitrogen gas sorption isotherms at -196°C and (b) pore size distribution of pristine DLC Supra 30 derived by use of the quenched solid density functional theory and by assuming slit-shaped pores. The average pore size was obtained from the cumulative pore volume distribution. SPT: standard temperature and pressure.


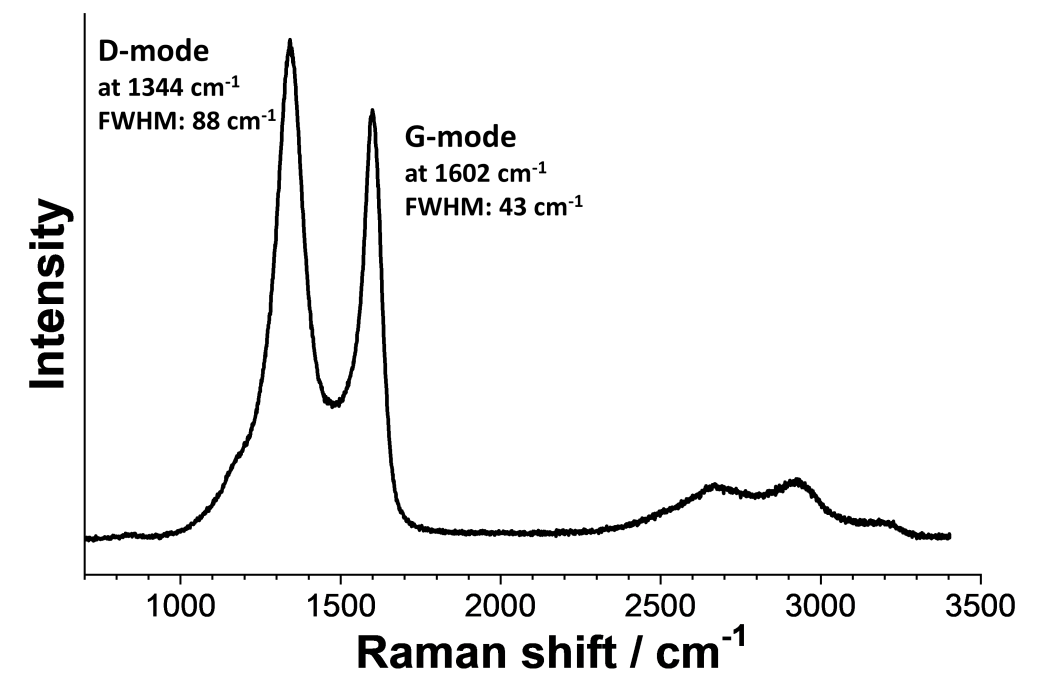


**Figure S5**. Raman spectrum of DLC Supra 30 at an excitation wavelength of 532 nm. The Raman spectrum was recorded using a numeric aperture of 0.75 and a laser power of ca. 0.2 mW at the focal spot of the sample with an inVia Raman spectrometer from Renishaw.


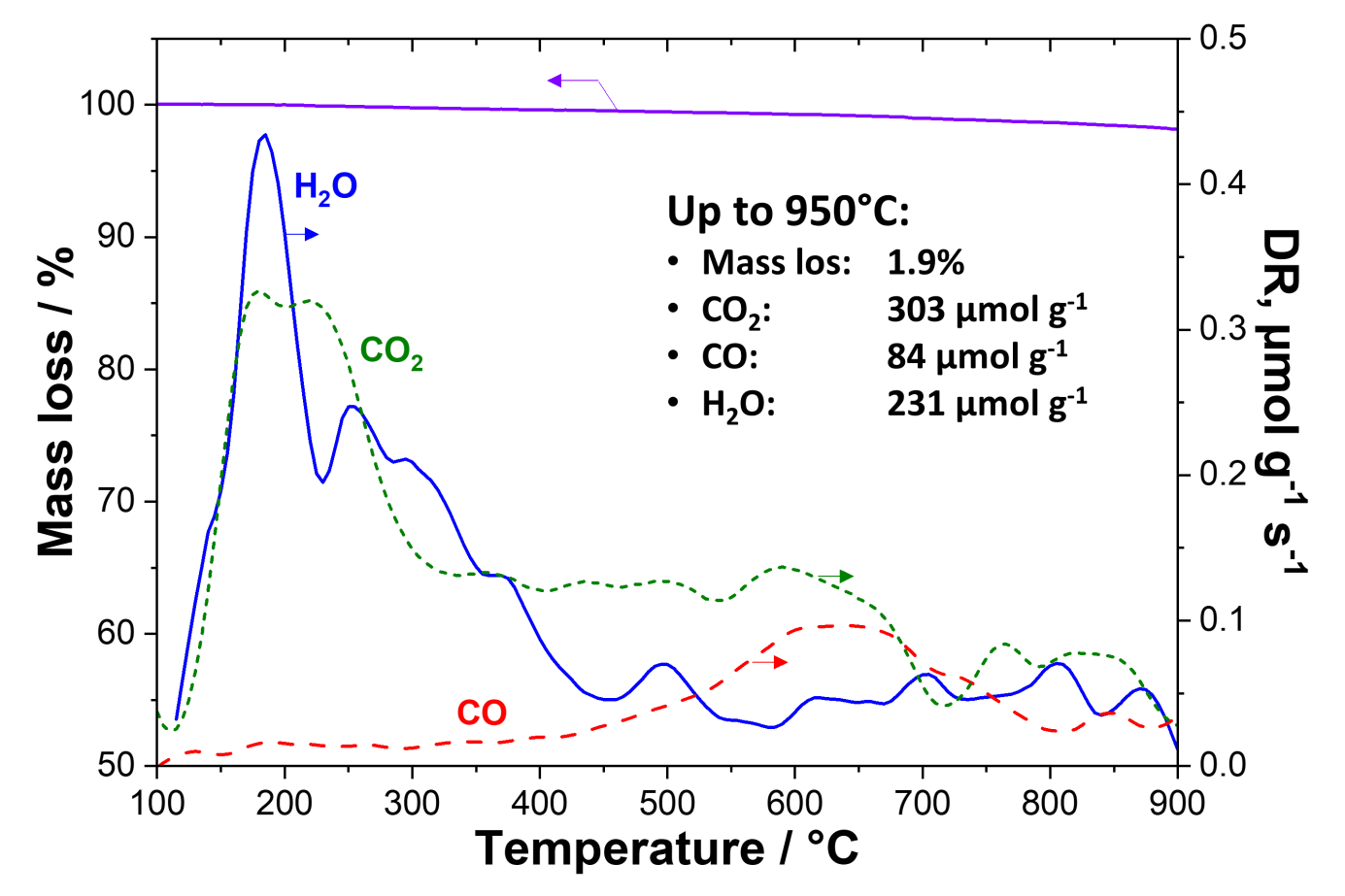


**Figure S6**. Thermogravimetric (TG) curve of DLC Supra 30 and CO, CO_2_, and H_2_O desorption rates during temperature-programmed desorption. The measurements were performed with a TG209 F1 Iris and Aëolos QMS 403C system from Netzsch.
